# *Gottschelia* (Gottscheliaceae, Marchantiophyta) in Indochina

**DOI:** 10.3390/plants13162198

**Published:** 2024-08-08

**Authors:** Vadim A. Bakalin, Anna A. Vilnet, Ksenia G. Klimova, Van Sinh Nguyen, Seung Se Choi

**Affiliations:** 1 Laboratory of Cryptogamic Biota, Botanical Garden-Institute of the Far Eastern Branch of the Russian Academy of Sciences, Makovskogo Street 142, Vladivostok 690024, Russia; ksenia.g.klimova@mail.ru; 2 Polar-Alpine Botanical Garden-Institute of the Russian Academy of Sciences, Akademgorodok 18A, Apatity 184209, Russia; anya_v@list.ru; 3 Institute of Ecology and Biological Resources, Graduate University of Science and Technology, Vietnam Academy of Science and Technology, Hanoi 10072, Vietnam; vansinh.nguyen@iebr.ac.vn; 4 Team of National Ecosystem Survey, National Institute of Ecology, Keumgangro 1210, Seocheon 33657, Republic of Korea

**Keywords:** *Chaetophyllopsis*, *trn*G-intron, *trn*L-F, *rbc*L, ITS1-2, Southeast Asia, Vietnam, taxonomy, liverworts, geographical species concept

## Abstract

*Gottschelia*, collected for the first time in Indochina, inspired an attempt to review the genus phylogeny to identify a more precise position of Indochinese plants. The genetic distance between African and Asian populations of *G. schizopleura* sensu lato was confirmed. The two groups should be treated as different species. A new combination, *G. microphylla* comb. nov., has been proposed for Asian plants. Aside from molecular genetics, distinguishing this species from the presumable strictly African *G. schizopleura* is also possible by morphological characteristics, as well as by its distribution. At the same time, at least three groups are distinguished among Asian haplotypes of *G. microphylla*, each of which can be interpreted as a species or, at least, subspecies. A morphological description, intravital photographs of the general habitat, and details of the morphological structures are provided. The position of *Gottschelia* in the phylogenetic schema of Jungermanniales does not allow us to attribute it to any of the known families and forces us to describe a new family, Gottscheliaceae, which is phylogenetically somewhat related to the Chaetophyllopsidaceae re-evaluated here and very different from Gottscheliaceae morphologically.

## 1. Introduction

*Gottschelia* is a bispecific paleotropic genus of liverworts, described more than 55 years ago by Grolle [1]. It was initially annotated by Grolle [1] as being morphologically similar to *Anastrophyllum*, *Hattoria*, *Herzogobryum*, and *Jamesoniella*. Even from these genera, the first two of which belong in the modern interpretation [2] to Anastrophyllaceae, the third to Cephaloziellaceae s.l. (in the ‘superfamily’ concept, following [3]), and the fourth to Adelanthaceae, it is clear that the position of the genus in the general phylogeny is at least debatable. In one of the early molecular phylogenetic studies, *Gottschelia schizopleura* (based on a specimen from Reunion) was found to have affinity for the genus *Lophonardia* [4]. A further precise study by Feldberg et al. [5] suggested that African and Asian accessions of *Gottschelia schizopleura* are sisters to *Chaetophyllopsis whiteleggei*, which belongs to its own morphologically distinctive family, Chaetophyllopsidaceae, with only two genera, *Chaetophyllopsis* and *Herzogianthus* [6]. The latter family, based on molecular studies, has been suggested to be polyphyletic: *Chaetophyllopsis whiteleggei* is phylogenetically related to some genera of Scapaniaceae sensu lato, and *Herzogianthus vaginatus* is placed in its own phylum, branched below Ptilidiales [7]. Later, *Gottschelia* and *Chaetophyllopsis* were classified in the family Scapaniaceae [8], and *Herzogianthus vaginatus* was segregated in its own family, Herzogianthaceae, in the order Ptilidiales [9]. Subsequent phylogenetic relationships of *Gottschelia schizopleura* with *Chaetophyllopsis whiteleggei* were demonstrated by Pazak et al. [10], who treated *Gottschelia schizopleura* as a member of Scapaniaceae, and by Katagiri and Inoue [11], who treated it within the complex family Cephaloziellaceae s.l. [3]. The relation of *Gottschelia* to the genus *Andrewsianthus* was determined by Feldberg et al. [12], and that to the genus *Anastrophyllopsis* was determined by Shaw et al. [13] (both genera are currently treated as members of Cephaloziellaceae s.l.). Subsequently, the affinities of *Gottschelia* and *Chaetophyllopsis* for *Andrewsianthus* and *Anastrophyllopsis* were shown by Bakalin et al. [14]. Therefore, to date, *Gottschelia* does not belong to the family Scapaniaceae sensu stricto. However, its placement in clearly polyphyletic and morphologically heterogeneous Cephaloziellaceae (as was also mentioned by Vana et al. [3]) is artificial as well. The present account provides a new attempt to identify the phylogenetic position of *Gottschelia* within the scope of currently available material.

It is worth mentioning that the controversial morphology of *Gottschelia* led to the description of two more taxa (*Gottschelia grollei* and *G. patoniae*) within the genus, and after molecular comparison, these taxa were subsequently transferred to the genus *Solenostoma* [5]. Therefore, to date, *Gottschelia* is regarded as a bispecific genus (the only species are *Gottschelia schizopleura* and *G. maxima*) with a rather unclear family placement. The genus *Gottschelia* was not previously reported from Indochina, so when we discovered plants belonging to this genus at the northern tip of the Annamite Range (an axial structure stretching 1200 km sub-meridionally across almost the entire Indochina Peninsula), we became interested in identifying the specific identity of the collected plants and attempted to analyze the phylogenetic relationships of this genus. The new input to solving both problems is the main goal of the present account.

## 2. Results

### Molecular Phylogenetic Reconstruction

**Figure 1:**
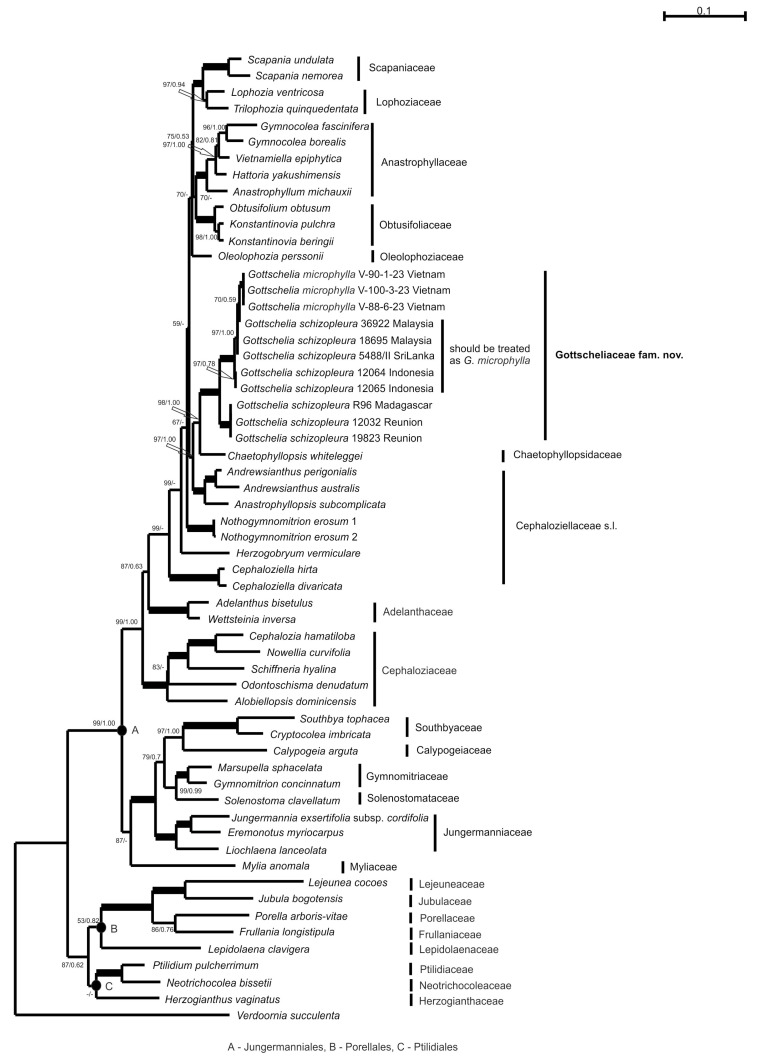
The phylogram for the Jungermanniidae obtained from the maximum likelihood approach based on *rbc*L + *trn*L-F cpDNA. Bootstrap support values and posterior probabilities more than 50% (0.50) are indicated. The branches stretched from nodes with 100% bootstrap support values and 1.00 posterior probabilities are in bold. A—order Jungermanniales, B—order Porellales, and C—order Ptilidiales.

The nucleotide sequences of *rbc*L, *trn*L-F, and *trn*G-intron and the separately assembled ITS1 and ITS2 sequences were obtained for each of three tested Vietnamese *Gottschelia* specimens and deposited into GenBank, as well as the ITS2 sequence for *Gottschelia schizopleura* from Tanzania (#68). The produced and phylogenetically tested alignment of combined *rbc*L and *trn*L-F nucleotide sequence data included accessions from 59 specimens. The dataset includes 1830 positions, among which 1124 positions belong to *rbc*L and 706 to *trn*L-F. The arithmetic mean of log likelihood in the ML calculation was −17,028.831, and in the two runs of the BA analysis, the values were −16,789.51 and −16,791.55. The topologies from both estimations are congruent, and Figure 1 illustrates an ML tree with bootstrap support (BS) values from ML analyses and Bayesian posterior probabilities (PP) from BA. As expected, the constructed topology agreed with that previously obtained and did not contradict the commonly accepted leafy liverwort’s phylogeny [15].

The families combined into three lineages corresponding to three orders: Jungermanniales (clade A), Porellales (clade B), and Ptilidiales (clade C). *Herzogianthus vaginatus* was found in the clade Ptilidiales (C) but without support, which confirmed its segregation from Chaetophyllopsidaceae in the distinct family Herzogianthaceae in the order Ptilidiales [9]. Other taxa in focus were placed in the clade of the order Jungermanniales (A). The families Scapaniaceae, Anastrophyllaceae, and Obtusifoliaceae composed robustly supported clades (BS = 100% in ML and PP = 1.00 in BA or 100/1.00), and the clade of Lophoziaceae was supported by 97/0.94. The sister relation of Scapaniaceae and Lophoziaceae achieved the highest support at 100/1.00, whereas the affinity of Anastrophyllaceae with the recently described family Obtusifoliaceae received poor support only on ML estimation (70%). The position of Oleolophoziaceae was also poorly supported (70%). The family Cephaloziellaceae s.l. had clear polyphyly with four subsequently diverged linages. The first linage (99/-) was represented by the genus *Cephaloziella*, the second (99/-) by the genus *Herzogobryum*, the third (67/-) by the genus *Nothogymnomitrion*, and the fourth by sister-related clades (97/1.00) of the genera *Andrewsianthus* + *Anastrophyllopsis* (100/1.00) and *Chaetophyllopsis* + *Gottschelia* (98/1.00). The specimens of the genus *Gottschelia* (100/1.00) were distributed in two robustly supported subclades according to their geographical origin—Africa and Asia.

The nucleotide sequence divergence between the African and Asian subclades of the genus *Gottschelia* was 1.9–2.1% for *rbc*L (Table 1).

The specimens from Madagascar and Reunion were characterized by sequence identity, whereas in the Asian subclade, nucleotide sequences of *rbc*L varied by 0.3–0.9% between different localities. At the same time, all Vietnamese specimens possessed identity at all five sequenced loci, and the *rbc*L gene sequences of specimens from Malaysia and Sri Lanka were identical to those of specimens from Indonesia. The divergence in *trn*L-F (0.7%) was marked only between Vietnamese specimens and specimens from Malaysia (#36922). The divergence in *trn*G-intron between Vietnamese specimens and specimens from Reunion (#15883) was 6.0%. The difference in nuclear ITS2 between Vietnamese and Tanzanian (#68) specimens reached 11.0%.

**Figure 2:**
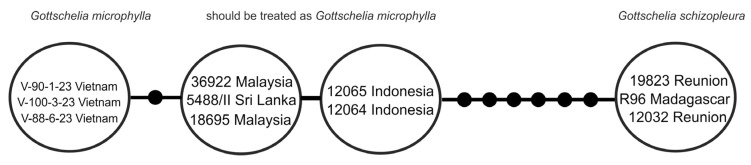
Statistical parsimony network for the *rbc*L cpDNA (451 base pairs) for the genus *Gottschelia*. Solid points indicate missing haplotypes. The specimen vouchers, according to Table 2, are marked.

The haplotype analysis of part of the *rbc*L gene revealed the presence of four haplotypes in the analyzed *Gottschelia* specimens (Figure 2). The Madagascar and Reunion specimens possessed a single haplotype. The Asian specimens were combined into three slightly diverged haplotypes that corresponded with geographical regions: Vietnam, Malaysia + Sri Lanka, and Indonesia. The distances between the African and three Asian haplotypes significantly exceeded the distances among the Asian haplotypes.

**Figure 3:**
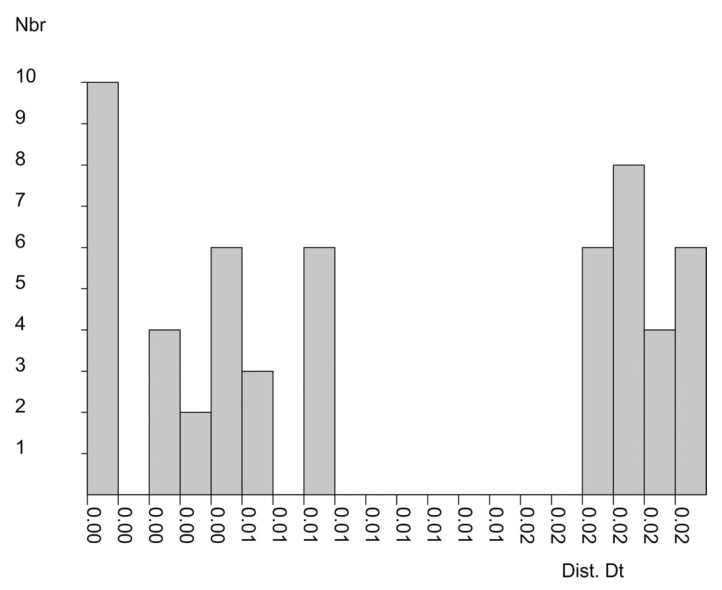
The distribution of pairwise *p*-distances between *rbc*L sequences (451 b.p.) of the genus *Gottschelia* specimens. Dist. Dt—distance value, and Nbr—number of runs.

The *p*-distances calculated for the *rbc*L sequences of the specimens from the genus *Gottschelia* via ASAP analysis were divided into four groups, separated by three gaps (Figure 3). The two groups of *p*-distances that corresponded to the separation of African and Asian accessions were clearly different. The less pronounced segregation of *p*-distances from Asian accessions corresponded with lower sequence divergence within this geographical region.

Thus, Cephaloziellaceae, in its broad sense, suggested by Vana et al. [3], is polyphyletic (which also corresponds to previous papers), and its splitting into smaller entities is likely necessary to continue. Despite the fact that *Chaetophyllopsis* and *Gottschelia* are sisters among all of the variants of topologies, the morphological dissimilarity and high genetic distances did not allow a simple re-evaluation of Chaetophyllopsidaceae and embedding of *Gottschelia*. In contrast, we suggest the description of a new family for *Gottschelia*.

The second result of the analysis was the confirmed significant genetic distance between the African and Asian (in a broad sense, including Malesia and Melanesia) populations of *Gottschelia schizopleura*, which should be interpreted as different species. Moreover, within the Asian complex, there are at least three units that should be described as species; however, this is currently not possible due to limited data.

## 3. Discussion

### 3.1. Molecular-Genetic Estimations

Following the transfer of *Gottschelia grollei* and *G. patoniae* to *Solenostoma* s.l. by Feldberg et al. [5], the genus *Gottschelia* became bispecific, including *G. maxima* characterized by a crenulate leaf margin (and otherwise quite similar to *G. schizopleura*) and *G. schizopleura* sensu lato. The latter taxon was interpreted very broadly, as shown by our obtained results and as also noted by the authors of the mentioned paper [5]. Both studies noted high genetic dissimilarity between African and Asian–Melanesian populations. Feldberg et al. [5] (p. 249) wrote “The African and Asian accessions of *G. schizopleura* are separated [in phylogenetic trees] by long branches (Figure 2) [in l.c.], indicating a significant period of isolation of the respective populations”, and further, “The Madagascar and Reunion accessions of *G. schizopleura* are more robust than the accessions from Indonesia, Malaysia and Sri Lanka”.

The median age of *Gottschelia* origin was estimated to be 34.65 Ma by Laenen et al. [16]. These ages were similar for the genera *Mylia* (35.7 Ma), *Liochlaena* (32.54 Ma), and *Hygrobiella* (31.36 Ma) [16]. The sequence divergence between species of the genus *Mylia* was 4.1–6.0% for *trn*L-F [17], 0.7–0.9% for *Liochlaena* for *trn*L-F and 0.9–1.6% for *trn*G-intron [18], and 2.1–3.6% for *Hygrobiella* for *trn*L-F and 2.2–3.0% for *trn*G-intron [19]. The divergence between the Reunion and Vietnamese specimens from the *trn*G-intron was quite high (6.0%) and could be treated as an additional differentiated feature to recognize them as distinct species.

### 3.2. Taxonomy

Our Vietnamese materials were genetically clearly placed within the broadly Asian macro-clade and, therefore, one cannot avoid discussing the question of the taxonomic status of Asian populations here. We studied the morphology of several specimens from Asian populations and only one specimen from Africa (see the Specimens Examined Section). In addition, we used the morphological data provided by Mufeed and Manju [20]. Overall, the materials reviewed supported the observations of Feldberg et al. [5]. All plants from Asia + Melanesia were characterized by the following: (1) rigid plants, versus soft and relatively lax plants in Africa; (2) smaller sizes, reaching only 1.5 mm (although it should be noted that materials from Vietnam were much larger than other Asian materials, up to 2.5 mm-wide and approaching the size of the African specimen); (3) the leaf sizes in Asian materials varied within 1.0–1.4 × 1.0–1.6 mm, except for Vietnamese materials, where they were the same size as African ones: 1.35–1.75 × 1.5–2.0 mm; (4) leaf cells in the mid-leaf in Asian materials were shortly oblong and varied near 20–30 × 20–25 µm, versus usually sub-isodiametric, 25–33 µm in diameter, in the African population; (5) the size of the cells in the lower part of the leaf in both groups overlapped, but were more elongated in Asian populations, 30–47 × 20–30 µm, versus 30–50 × 24–30 µm in African populations; (6) the cuticle was shortly papillose (not striolate) in the African population, versus mostly papillose-striolate in Asian populations; (7) the most clear difference was observed in the shape of the perianth, which was fusiform in Asian populations (that is, gradually becoming wider and then gradually narrowing), while in African plants, the perianth was rather cylindrical, rather abruptly narrowed toward the mouth; (8) a significant difference was also observed in the comparative length of the thin-walled cell zone in the upper part of the perianth, which constituted up to 1/4 of the perianth length in the Asian populations and only about 1/15–1/20 in African populations; (9) cells in the lower quarter of the perianth were shorter and wider in the African population, 30–50 (60) × 28–35 µm, and nearly thin-walled without intermediate thickenings, versus 50–75 × 12–18 µm with nearly thin walls, but with numerous intermediate thickenings in Asian populations; (10) the leaf insertion line in African populations was laterally merely oblique, while it was clearly transverse in Asian populations.

According to the available data, the Vietnamese populations represented the largest “phases” of the Asian taxonomic entity, which differed from the African taxon in a number of the above-listed characters. Considering its genetic differences, this Asian taxon should be considered an independent species and should be named *Gottschelia microphylla* comb. nov., described as *Jungermannia colorata* var. *microphylla* by Nees [21]. At the same time, it is worth mentioning that the Asian group itself may also split into several species, taking into account the genetic distances between the subclades, and the Vietnamese populations may well deserve to be treated as separate species characterized by larger sizes of plants, leaves, and cells. Other regional populations may also be different taxa; if not species, then subspecies. For example, a specimen from Papua New Guinea (Sleumer & Vink 4316) does not show purple–brown pigmentation, which is characteristic of other specimens (noticeably, purple pigmentation was mentioned as a generic characteristic in the original description of Grolle) [1]. In this paper, we currently accepted the species status of Asian populations but did not identify Vietnamese plants as a separate taxon, which requires an extensive study of not only fresh material using molecular genetic methods but also a restudy of type materials of at least the following names: *Jamesoniella fleisherii* Steph., *Anastrophyllum cucullifolium* Steph., and *A. integerrimum* Steph., listed by Grolle in the list of synonyms of *Gottschelia schizopleura*. The status of *Gottschelia maxima* also remains unresolved. Our attempt to obtain the sequence of the specimen of the latter species failed, as in [5]. The morphology of plants in the studied specimen of *G. maxima* (see the Specimens Examined Section) in terms of the size of leaf cells and the papillose cuticle feature was quite close to that of the African plant *Gottschelia schizopleura*, but there were clear differences in the nature of the leaf margin.

Below, we present a description of *Gottschelia microphylla* based entirely on materials from Vietnam and provide new combination proposals. In future research, the ‘Asian’ clade of *Gottschelia*, named here as *G. microphylla*, could be split into narrower taxa, and then the name *G. microphylla* could not be applied to Vietnamese plants. To avoid the artificial mixture of morphological features, we did not provide a unified description based on *Gottschelia* materials collected in various parts of Asia.

*Gottschelia microphylla* (Nees) Bakalin, Vilnet, Klimova et S.S. Choi comb. nov.

Basionym: *Jungermannia colorata* var. *microphylla* Nees Enumeratio Plantarum Cryptogamicarum Javae 27. 1830.

**Figure 4:**
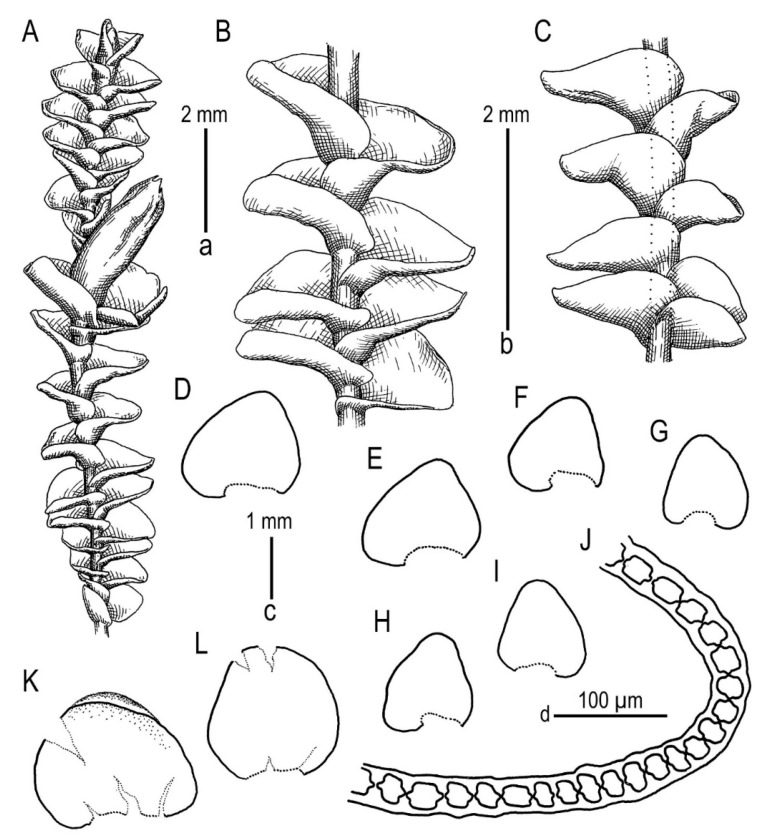
*Gottschelia microphylla* (Nees) Bakalin, Vilnet, Klimova et S.S. Choi: (**A**) Perianthous shoot, dorsal view. (**B**) Part of shoot, dorsal view. (**C**) Part of shoot, ventral view. (**D**–**I**) Leaves, (**J**) perianth cross-section in its middle part, and (**K**,**L**) female bracts. Scales: a—2 mm for (**A**), b—2 mm for (**B**,**C**), c—1 mm for (**D**–**I**,**K**,**L**), and d—100 µm for (**J**). All from V-88-15-23 (VBGI, HN).

**Figure 5:**
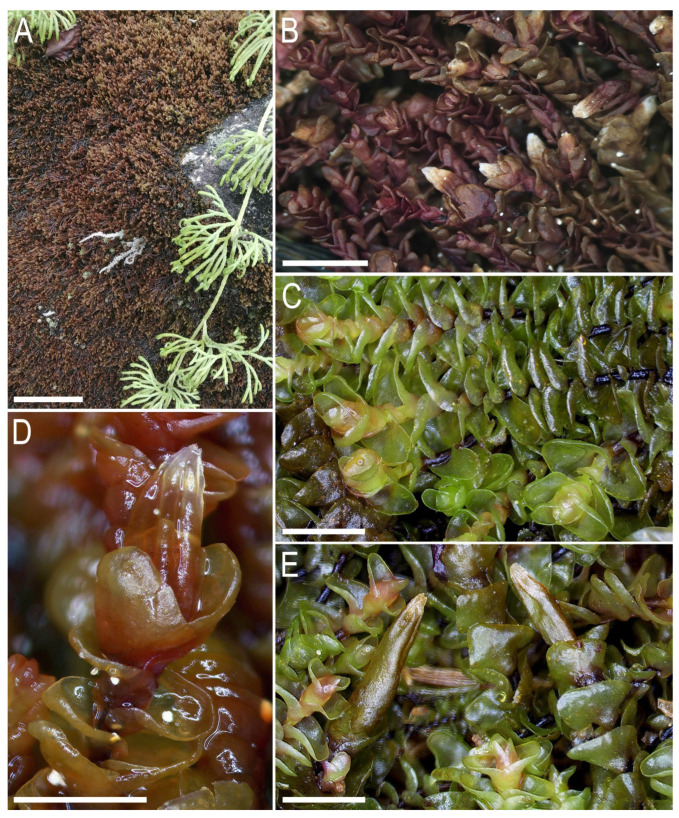
*Gottschelia microphylla* (Nees) Bakalin, Vilnet, Klimova et S.S. Choi: (**A**) Mat of *Gottschelia microphylla* covering rocks in natural conditions. (**B**) Mat with perianthous shoots in dry condition. (**C**) Shoots, dorsal view. (**D**) Fragment of perianthous shoot, dorsal view. (**E**) Perianthous shoots in a mat, dorsal view. Scales: 5 mm for (**A**,**B**) and 2 mm for (**C**–**E**). (**B**,**D**) From V-88-15-23, and (**C**,**E**) from V-88-7-23 (VBGI, NH).

**Figure 6:**
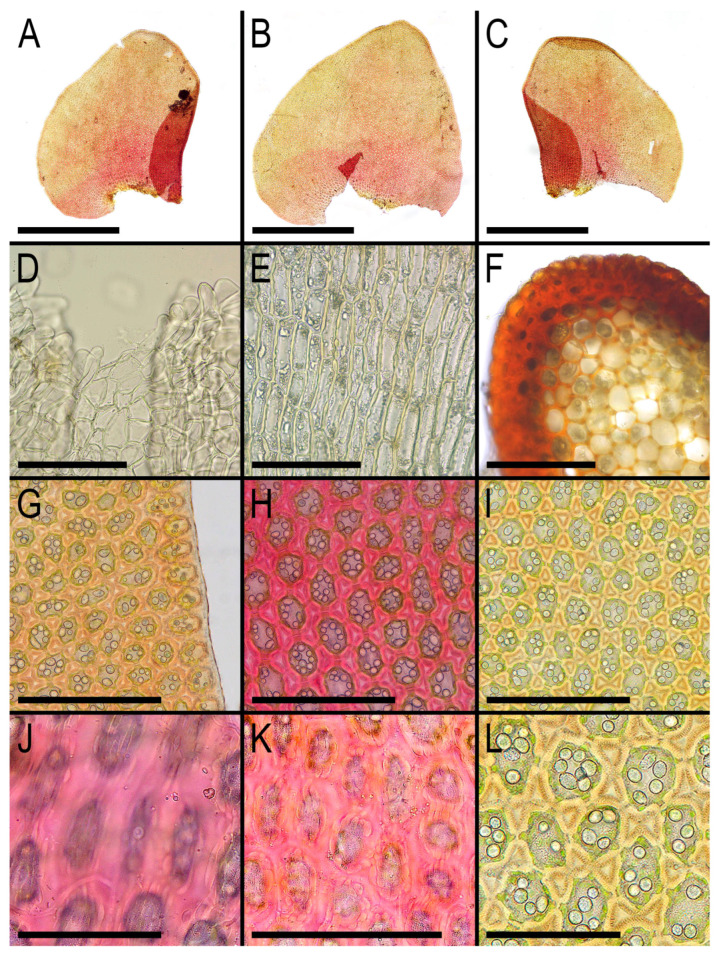
*Gottschelia microphylla* (Nees) Bakalin, Vilnet, Klimova et S.S. Choi: (**A**–**C**) Leaves, curled up and torn under the slide. (**D**) Perianth mouth armature. (**E**) Perianth cells in lower part. (**F**) Stem cross-section, fragment. (**G**) Leaf cells along leaf margin. (**H**,**I**,**L**) Mid-leaf cells with oil bodies. (**J**) Papillose cuticle in lower part of the leaf. (**K**) Papillose cuticle in the mid-leaf. Scales: 1 mm for (**A**–**C**), 3 mm for (**F**), 100 µm for (**D**–**K**), and 50 µm for (**L**). (**A**–**F**,**I**–**K**) From V-88-15-23, and (**G**,**H**) from V-88-6-23 (VBGI, NH).

Description (based on Vietnamese materials). Plants were rigid, erect to ascending, in loose pure patches or mixed with other liverworts, brown–purple to wine–brown, 2.0–2.5 mm-wide (to 3 mm near perichaetia), 20–30 mm-long, with older parts decaying. Rhizoids were not seen. Stem brown to blackish brown, nearly straight, and branching was seen only as ventral sub-floral innovation. Cross-section was suborbicular to slightly transversely ellipsoidal, with 3 (–4)-stratose well-defined cortex composed by cells 12–18 µm in diameter, with strongly thickened walls (walls were sometimes so strongly thickened that the cell lumen collapsed), visible middle lamina, and outer wall papillose-striolate to nearly smooth. Inward cells suddenly became thin-walled or with only slightly thickened walls, 15–25 µm in diameter, 5–6-gonal, with small concave trigones. Leaves were obliquely spreading, somewhat or distinctly turned dorsally, distinctly canaliculate with margins turned inward (incurved adaxially), loosely sheathing the stem in the lower third, inserted transversely, with insertion lines turned up to the apex, both in dorsal and ventral ends. Flattened in the slide, commonly torn or plicate, ovate to widely ovate-triangular, 1350–1750 × 1500–2000 µm. Mid-leaf cells were sub-isodiametric to shortly oblong, 25–30 × 22–25 µm, with angular, very large trigones, abruptly turning to thin walls. Trigones had well-perceptible middle lamina with small transverse lines (similar to small plasmodesmata), and the cuticle was loosely to distinctly papillose. Cells along the leaf margin in the lower part of the leaf had a strongly thickened external wall, subquadrate to rectangular (then elongated perpendicularly to the leaf margin), 12–20 × 12–25 µm. Other walls were thin, but with very large angular trigones with well-visible middle lamina, which seemed never or very rarely confluent. Cells along the margin in the upper part of the leaf were similar to those in the lower margin, ca. 20–25 µm in diameter, with trigones sometimes confluent, and the cuticle loosely papillose. Cells in the lower 1/4 of the leaf length were oblong, 30–47 × 20–30 µm, with trigones similar to the mid-leaf, and the cuticle scarcely papillose. Under-leaves were absent. Dioicous (male plants not seen). Female bracteole were absent. Female bracts were widely ovate-triangular, 2200–2300 × 2200–2750 µm, with a margin entirely or somewhat crispate, with a sometimes-discolored distal margin. Perigynium was absent. Perianth fusiform, 3.0–4.0 × 1.0–1.2 mm, pluriplicate, except the lower third, gradually narrowed to narrow, but not beaked mouth. Mouth denticulate along the margin. Perianth cells were thin-walled with small concave trigones in the upper 1/4 of its length, then became thin-walled with angular and noticeable large trigones, similar to leaves in the perianth middle, and then became strongly oblong in the lower 1/4 of the perianth length, 50–75 × 12–18 µm, with nearly thin walls, but with numerous intermediate thickenings. Perianth unistratose. Archegonia 15–20 per perichaetium (always unfertilized in our specimens; Figure 4, Figure 5 and Figure 6).

Specimens examined:

*Gottschelia maxima* Papua New Guinea, Morobe Province, Kaindi Mt. (estimated coordinates 7.33229° S, 146.68166° E), 3000 m a.s.l., montane rainforest, 19 June 1984, B. Theirs 3299 (VBGI), originally identified as *G. crenata* Grolle.

*Gottschelia microphylla* Vietnam, North Central Vietnam, Nghệ An Province, Kỳ Sơn District, Na Ngoi Commune, Annamite Range (19.22636° N 104.11698° E), 1826 m a.s.l., the road across evergreen forest on steep slope, open moist cliff along the stream, 8 June 2023, V.A. Bakalin & M.H. Nguyễn V-87-3-23 (VBGI, HN); ibid. V.A. Bakalin & M.H. Nguyễn V-87-8-23 (VBGI, HN); ibid. (19.22826° N 104.11379° E), 1871 m a.s.l., the road across evergreen forest on steep slope, open moist cliff, 8 June 2023, V.A. Bakalin & M.H. Nguyễn V-88-6-23 (VBGI, HN); ibid. partly shaded moist cliff., 8 June 2023, V.A. Bakalin & M.H. Nguyễn V-88-7-23, V-88-15-23 (VBGI, HN); ibid. (19.22950° N 104.10697° E), 1838 m a.s.l., the road across evergreen forest on steep slope, open moist cliff, 8 June 2023, V.A. Bakalin & M.H. Nguyễn V-90-1-23 (VBGI, HN); ibid. (19.23096° N 104.11337° E), 1779 m a.s.l., the road across evergreen forest, open moist clayish road cut, 10 June 2023, V.A. Bakalin & M.H. Nguyễn V-100-3-23 (VBGI, HN)/.

*Gottschelia schizopleura* MALAYSIA, Pahang State, Titiwangsa Range, Cameron Highlands, Batu Brinchang Mt. (Gunung Batu Brinchang) (estimated coordinates 4.52096° N, 101.38249° E), 1950 m a.s.l., roadside bank, 19 October 1967, N. Kitagawa NY: 14,737 (VBGI); ibid. Genting Highlands, Ulu Kali Mt. (Gunung Ulu Kali) (3.41667° N 101.78333° E), 1700 m a.s.l., on soil bank of roadside, 8 November 1999, M. Higuchi, No.1221 in Bryophyta Selecta Exsiccata. Fasc. XXIX, Higuchi (2008) (VBGI); ibid. Mt. Brinchang, near Brinchang (4.51667° N 101.36667° E), 1550 m a.s.l., on bare soil at roadside bank, 29 July 2007, M. Higuchi No. 1443 in Bryophyta Selecta Exsiccata. Fasc. XXXVIII, Higuchi (2017) (VBGI); Tanzania, Moshi District, Kilimanjaro Mts., north of the village of Kibosho (3.23333° S 37.31667° E), 1950 m a.s.l., degraded montane rainforest above Osaki Forest Station, with scattered Macaranga, Agauria, Ocotea, and Myrica, on roadcut surface, 19 October 1967, T. Pocs, J.S. Miala & P.P. Maragesi 90066/D, No. 68 in Bryophyta Africana Selecta Series III, Ochyra, Pocs (1992) (VBGI); Indonesia, Western New Guinea, Pegunungan Arfak Regency, Arfac Mt., Pegunungan Arfak Nature Reserve (estimated coordinates 1.156295° S, 133.97962° E), 3000 m a.s.l., 1962, Sleumer & Vink 4316 (VBGI).

### 3.3. Distribution and Ecology

The Asian–Australasian range of *Gottschelia microphylla* extends from South India and Sri Lanka through the Philippines, Malaysia, and Indonesia to the Moluccas and Papua New Guinea. The northernmost outpost of the species is in Taiwan [22]. Indochina is, therefore, considered the missing link in this list of regions, and the discovery of *Gottschelia* here should have been expected. This genus was discovered in Indochina in Vietnam on the Annamite Range, a little-studied system extending from the northern part of Laos and Vietnam (separating them) to almost the plain of the Mekong River in southern Vietnam.

The Annamite Range is widely known as the focus for the concentration of a unique and very rich flora and fauna in Indochina, in part due to its sub-meridional stretching, connecting the tropical rainforests of the southern tip of Indochina with the mountainous subtropical-temperature vegetation of the foothills of the Hengduan mountain system, including the Hoang Lien Son Range [23,24,25,26,27,28,29,30]. Little is known about the liverworts of the Annamite Range, although information has continued to accumulate in recent years, for example, due to efforts by Pocs et al. [31,32]. *Bazzania asperrima* Steph. was described from the Annamite Range in the past [33]. *Gottschelia microphylla* is another unique element of the Annamite Range biota, along with numerous other examples cited in the above literature sources.

As noted, when describing the genus [1], *Gottschelia schizopleura* s.l. grows mostly on bare soil and is found much less often on stones and tree trunks. The same habitat span was subsequently reported in the literature. All known locations of the species are restricted to the upper mountain belts. The species rarely descends even to rainforests at middle elevations. Apparently, the ecology of African *Gottschelia schizopleura* and Asian *G. microphylla* is similar, considering the studied specimens and literature data [1,20,22]. Interestingly, the northernmost record of *G. microphylla* in the world was made on the wet bark of a living tree in a mossy forest in Taiwan [22], but not on bare soil. All of our Vietnamese collections of the species were performed between 1779 and 1871 m a.s.l.; that is, in a rather narrow altitudinal range, not going lower (which is quite understandable, given that anthropogenically transformed habitats begin quite soon below), but not going higher either, although the altitudinal range in the study area extended to 2720 m a.s.l., as far as the top of Mt. Phu Xai Lai Leng, which we also studied. Moreover, most of the Vietnamese collections are associated with the open ground of anthropogenic origin (talus along roads), although the species is also found in the studied area on presumable granitic rocks along streams (but outside the zone of direct washing of flowing water). All collections were performed in open and temporarily moist, but likely often very dry places, so the xerophytic morphology of the species (papillose cuticle, large trigones in the leaf cells) appears very reasonable in this context. In terms of ecology, the species is somewhat reminiscent of *Jackiella javanica* and *Plectocolea hasskarliana*, which often grow together with *Gottschelia microphylla* in Vietnam, having not only a similar ecology but also a similar (albeit wider) range. Considering the neutral reaction of the sandstone rocks at the study site, the species can generally be characterized as a neutrophylous meso-xerophyte. The bioclimatic data (Table S1) showed the following: (1) a warm climate with an annual mean temperature of 16–17 °C, (2) seasonal temperature variation that is not strong and decreases to 12 °C in the coldest quarter but reaches 19–20 °C in the warmest quarter, (3) a distinct monsoon climate with strong precipitation variation across the year, with values of 260–270 mm in the wettest month and only 4–6 mm per driest month, and (4) a total amount of precipitation reaching 1800–1900 mm per year. A striking feature of the studied area is the high probability of sporadic snowfalls in winter. The referenced area (https://phapluatplus.vn/tuyet-phu-trang-o-chan-nui-phu-xai-lai-leng-12632.html (accessed on 22 June 2024)) shows the site slightly above the location where *Gottschelia* was collected. This phenomenon of snowfall at low altitudes far south of the Tropic of Cancer seems to be unique in Indochina.

### 3.4. The Position of Gottschelia within the Cephaloziellaceae–Scapaniaceae Superclade

*Gottschelia* specimens are rarely included in phylogenetic reconstructions, as reviewed in the Introduction. The first reliable phylogeny involving this genus was published by Feldberg et al. [5]. In the cited paper, *Gottschelia* has a sister position to *Chaetophyllopsis*, while *Chaetophyllopsis* first became involved in phylogenetic reconstructions by Hendry et al. [7], where it was also found in a sister position to Scapaniaceae s.l. The *Chaetophyllopsis* + *Gottschelia* pair is a remote sister to the Scapaniaceae s.l. superclade [5]. Based on the cited phylogeny, Váňa et al. [3] proposed that Scapaniaceae, Lophoziaceae, and Anastrophyllaceae should be considered in the traditional (=narrow) sense, while all genera ‘embedded’ between Scapaniaceae s.l. and Cephaloziellaceae s. str., including *Chaetophyllopsis* and *Gottschelia*, may be regarded as members of the ‘superfamily’ Cephaloziellaceae, which was distinctly polyphyletic in this case. The cited authors were fully aware that this position is partly artificial, and additional research is necessary to understand the system of the enormous clade of leafy liverworts Scapaniaceae s.l. + Cephaloziellaceae s.l. Later, a similar phylogeny was shown by Patzak et al. [10], but they interpreted it as distinctly different from that of Vana et al. [3] and treated Scapaniaceae in a very broad sense (then, it was graded to the ‘superfamily’) and maintained a narrower concept for Cephaloziellaceae.

We assume that both points of view are hardly convenient since either one or the other family (in this case, Scapaniaceae s.l. or Cephaloziellaceae s.l.) is also inconsistent in comparison with other accepted liverwort families. Our previous work described the small but genetically distinct families Obtusifoliaceae and Oleolophoziaceae [34,35]. This segregation process is clearly not complete, considering the large number of genera located between the finely defined clades, Cephaloziellaceae and Scapaniaceae–Lophoziaceae–Anastrophyllaceae, in known phylogenies. Since the assignment of *Gottschelia* to both Scapaniaceae and Cephaloziellaceae cannot be followed, it is necessary to find another family for this genus by restoring it from forgotten ones, if there are any, or by describing it as a new one.

Formally, the *Gottschelia* + *Chaetophyllopsis* clade has the name Chaetophyllopsidaceae for *Chaetophyllopsis*, and the simplest option is to restore the family Chaetophyllopsidaceae, which includes *Gottschelia*. However, this approach completely contradicts the morphology of both genera. It is difficult to imagine more morphologically dissimilar archetypes as *Chaetophyllopsis* and *Gottschelia*. Schuster [36] placed the isolated genus *Herzogianthus* morphologically close to *Chaetophyllopsis* (interpreted together with *Chaetophyllopsis* as representatives of the oligotypic Chaetophyllopsidaceae). *Herzogianthus* was later placed in its own family, Herzogianthaceae [9]. Both genera are characterized by leaf margins armed with stiff, hyaline, 1-celled setae (cilia), asymmetric succubous leaves and well-developed, basically bilobed under-leaves. Although there are several dissimilarities, e.g., in the presence of tetrahedrical 4–5-celled gemmae in *Chaetophyllopsis* (absent in *Herzogianthus*), leaves isomorphic, 3–4-lobed with some additional primary and several secondary lobes (versus leaves dimorphic, 3-lobed, without additional lobes and without secondary lobes), and rhizoids not confined to under-leaf bases (versus rhizoids confined to under-leaf bases). Several distinctions are also seen in the generative sphere, and they are perfectly described by Schuster [6]. Schuster [37] considered *Chaetophyllopsis* to be related to Ptilidiaceae and Lepidolaenaceae, but never expected the genus to be related to Scapaniaceae or something similar. In contrast, *Gottschelia* was suggested to be poorly related to *Anastrophyllum* [1]. Since *Anastrophyllum* in modern treatments belongs to the Scapaniaceae–Lophoziaceae–Anastrophyllaceae superclade, the suggestion by Grolle was relatively correct. Morphologically, *Gottschelia* and *Chaetophyllopsis* are strongly different in a number of features: unlobed versus lobed leaves (the same for female bracts) and absence versus the presence of under-leaves. However, some similarities may also be noted. They include the following: (1) brown to reddish-brown color of plants in both, (2) collenchymatous cell structure both in leaves and stem, (3) perianth fusiform with discolored mouth (although polystratose in the lower half in *Chaetophyllopsis*), and (4) 22 rows of outer cell walls in seta cross-section in both. Both genera also have stellate gemmae: unicellular in *Gottschelia* (not found in Vietnamese materials) versus 4-5-celled in *Chaetophyllopsis*. *Chaetophyllopsis* is an Australasian taxon, where the area of *Gottschelia* extends from Melanesia to the Southeast Asian mainland. Therefore, the two genera areas are somewhat in contact but not sympatric. Following the strong morphological dissimilarity below, we describe the new family to house *Gottschelia*.

Gottscheliaceae Bakalin, Vilnet, Klimova et S.S. Choi fam. nov.

Diagnosis. Plants were dioicous, rigid, and leaves were unlobed with entire or denticulate margin, concave-canaliculate, sheathing the stem at the base, and strongly interlocking dorsally. Under-leaves were absent. The cell network of leaves was pachydermous. Female bracteole were absent, and bracts were unlobed. Perigynium was absent. Perianth was fusiform, strongly exerted, pluriplicate near the mouth, and the upper part of the perianth was discolored, unistratose. Sub-floral innovation was present. Spores were verruculose. Elaters were bispiral. Gemmae were sometimes present, stellate, and unicellular.

Type genus: *Gottschelia* Grolle, J. Hattori Bot. Lab. 31: 13, 1968.

Included genera: The family is hitherto mono-generic.

The type species of the genus *Gottschelia*: *Gottschelia schizopleura* (Spruce) Grolle, J. Hattori Bot. Lab. 31: 16, 1968.

Basionym: *Jungermannia schizopleura* Spruce, Trans. & Proc. Bot. Soc. Edinburgh 15: 517, 1885.

The genus *Gottschelia* includes three species. Other included taxa are *Gottschelia maxima* (Steph.) Grolle and that evaluated above, *Gottschelia microphylla* (Nees) Bakalin, Vilnet, Klimova.

## 4. Materials and Methods

### 4.1. Materials and Nature Environment

The material used in this paper was collected during a survey of the northern end of the Annamite Range. The name Annam itself comes from the Vietnamese pronunciation of the Chinese ‘An Nan’, which has a literary meaning of ‘tranquil south’. This word was later used by the French during the colonial time of Vietnam, both to refer to the ridge and to the Vietnamese people in general. The Vietnamese themselves call the Annamite Range the Truong Son Range. This range, as noted above, has a sub-meridional strike of 1200 km and forms the orographic spine of all of Indochina. Administratively, the Annamite Range borders Laos and Cambodia from Vietnam. The highest point in the range is Mt. Phou Bia, which is located in Laos and reaches an altitude of 2819 m a.s.l. at its summit. The second highest mountain in the Annamite Range is Mt. Phu Xai Lai Leng, with a height of 2720 m a.s.l. This mountain is also located in the northern part of the range and is administratively divided in half between Laos and Vietnam. In Vietnam, it is administratively located in Vinh Province, which belongs to the North Central Vietnam group of provinces. This mountain was the object of our research in 2023, when *Gottschelia* was collected.

The Annamite Range is an ancient formation. The first and most powerful stage of uplift occurred during the Indonesian orogeny at the end of the Paleozoic–beginning of the Mesozoic era. At that time, a wide layer of marine sediments and underlying granitic intrusions were uplifted. At present, the study area consists of a few granite outcrops and mostly eroding sandstones dominating the mountains [30], while limestones are widespread at lower altitudes in the northern part of the Annam Range, but they are absent in the study area. The climate of the study area is a monsoon tropical climate associated with mountains, with annual precipitation varying between 2000 and 2500 mm per year [38]. The dominant type of vegetation is evergreen montane and highland forest on silicate rocks at 1000–3000 m a.s.l. Floristically, the study area belongs to the Northern Indochinese Floristic Province [30]. In phytogeographical terms, the northern part of the Annamite Range is interesting as an orographic route (or link) for mountain species common with the Sikang-Yunnan Floristic Province of the East Asian Floristic Region [39], sometimes treated as an East Asian Floristic Subkingdom or even kingdom [40] to the south, into subequatorial floras in southern Indochina.

The material for this work was collected during joint Russian–Vietnamese expeditions at the beginning of June 2023 to study the diversity of liverworts in the Annamite Range. When collecting specimens, geographic coordinates, altitude above sea level, type of vegetation community, and ecological habitat conditions, including moisture supply and illumination, were indicated. The specimens collected were studied at the Laboratory of Plant Ecology at the Institute of Ecology and Biological Resources of the Vietnam Academy of Science and Technology (Hanoi), where intravital *Gottschelia* photographs were taken using digital cameras mounted on Nikon SMZ800N and Olympus BX43 microscopes in the latter laboratory. During the initial study of the living material, some plants were stored in dry silica-gel-filled plastic zip lock bags for molecular genetic research. A detailed morphological description was subsequently compiled after the molecular genetic analysis.

Since the coordinates were known for all of our collected specimens in Vietnam, we obtained the bioclimates from WorldClim [41,42] (Table S1) for each point, which made it possible to compile the general climatic requirements for the obtained material. These data are listed in Table S1. To characterize each point, all 19 bioclimates were obtained, which could be determined with a maximum accuracy of 30″, and if the points were closer, then the parameters could be merged.

### 4.2. Molecular–Genetic Analysis

Three Vietnamese specimens morphologically identified as *Gottschelia* were included in the molecular phylogenetic estimation. The *rbc*L cpDNA was chosen as an appropriate molecular marker for the six sequenced accessions of *Gottschelia schizopleura* from [5], which were included in the current analysis. The *trn*L-F and *trn*G-intron were sequenced as additional markers to compare Vietnamese plants with specimens of *Gottschelia* from studies by Shaw et al. [13] and De Roo et al. [4]. The ITS1-2 nrDNA region was first sequenced in this study for specimens of *Gottschelia schizopleura* from Tanzania and three Vietnamese specimens to reveal the diversity in nuclear markers. The specimen vouchers and GenBank accession numbers for the specimens of Gottschelia sequenced in this study are listed in Table 2. For estimation, the accessions with different genetic regions of nine specimens of *Gottschelia schizopleura* were downloaded from GenBank. The unresolved relationship and questionable taxonomic position of the genus *Gottschelia* inspired us to analyze several key genera of Cephaloziellaceae s.l. previously estimated separately in different studies: *Chaetophyllopsis*, *Andrewsianthus*, *Anastrophyllopsis*, *Herzogobryum*, and *Nothogymnomitrion*. The dataset covered three orders: Jungermanniales, Porellales, and Ptilidiales, and *Verdoornia succulenta* from Metzgeriales was chosen as an outgroup following the general phylogeny in [15]. Additionally, 48 specimens, including those from recently segregated Obtusifoliaceae and Oleolophoziaceae, were downloaded from GenBank, 45 of which contained nucleotide sequence data for the *rbc*L and *trn*L-F loci. The list of downloaded specimens included in the molecular estimations with voucher details and GenBank accession numbers is provided in Table 3.

### 4.3. DNA Isolation, Amplification, and Sequencing

After being collected for molecular procedures, the liverwort plants were dried in silica gel. DNA was extracted with a DNeasy Plant Mini Kit (QIAGEN (Hilden, Germany)) following the manufacturer’s protocol. The pairs of primers suggested by Kress and Erickson [43] for *rbc*L, by Taberlet et al. [44] for *trn*L-F, by Shaw et al. [45] for *trn*G-intron, and by White et al. [46] for ITS1-2 were used for amplification and sequencing. PCR was carried out in 20 μL volumes with the following procedure: 3 min at 94 °C, 30 cycles (30 s at 94 °C, 40 s at 52 °C for *rbc*L, 56 °C for *trn*L-F and ITS1-2, 64 °C for *trn*G-intron, and 60 s at 72 °C), and 2 min for a final extension at 72 °C. The obtained amplicons were visualized on 1% agarose TAE gels by EthBr staining. Then, they were cleaned from agarose with a Cleanup Mini Kit (Evrogen, Moscow, Russia) and sequenced with an ABI Prism BigDye Terminator Cycle Sequencing Ready Reaction Kit (Applied Biosystems, Waltham, MA, USA) following the standard protocol provided for the 3730 DNA Analyzer (Applied Biosystems, Waltham, MA, USA) at the Genome Center of EIMB (Moscow, Russia).

### 4.4. Phylogenetic Analysis

The program BioEdit 7.0.1 [47] was used for sequence assembly and dataset alignment. The alignments of the *rbc*L and *trn*L-F datasets were automatically produced with the ClustalW tool and subsequently manually corrected. Preliminary estimations revealed a lack of incongruence from both DNA regions, and then *rbc*L and *trn*L-F were combined into a single dataset for subsequent analysis, with inclusion in the estimation of all positions and coding absent fragments as missing. Phylogeny was tested by maximum likelihood (ML) analyses with IQTree 1.6.12 [48] and the Bayesian approach with MrBayes v. 3.2.1 [49]. ModelFinder [50] selected TIM2 + F + I + G4 as the best-fit evolutionary model of nucleotide substitutions. The ultrafast bootstrapping procedure [51] included 1000 iterations. The ML tree topologies were visualized in NJplot [52]. The Bayesian calculation was run with the GTR + I+G model, as recommended by the developers of the program, with four rate categories of gamma distributions. Chains of two independent runs of the Metropolis-coupled ΜCMC were run for one million generations, and trees were sampled every 100th generation. The first 2500 trees in each run were discarded as burn-in, and 15,000 trees were sampled from both runs. The average standard deviation of the split frequencies between the two runs was 0.006454, and Bayesian posterior probabilities were calculated from the trees sampled after burn-in. The BA tree topologies were redrawn in FigTree v1.3.1 [53]. The level of nucleotide sequence variability among specimens of the genus *Gottschelia* from different geographical localities was estimated as average pairwise *p*-distances using the pairwise deletion option for counting gaps in Mega 11 [54]. The distribution of pairwise *p*-distances between *rbc*L sequences of the genus *Gottschelia* was determined by the ASAP program [55], with default settings and a *p*-distance model. The haplotype diversity of the genus *Gottschelia* was estimated in the TCS program [56] based on an analysis of *rbc*L alignment with a length of 451 base pairs common to all specimens.

## 5. Conclusions

As the present study showed, the genus *Gottschelia* was found in Indochina. This finding, considering the general distribution pattern of the genus, was quite expected. However, it did not concern the species name for the Vietnamese material, which is unclear. When considering the taxonomic position of the plants collected in Vietnam, it confirmed the earlier estimated high genetic and morphological differentiation of African and Asian+Papuasian plants, previously referred to as the same species (*G. schizopleura*). It was clearly shown that Asian plants cannot belong to the same species as African plants. Here, we were forced to search for a name for this taxon, which was carried out using a new combination of a previously existing name. However, this solution is somewhat preliminary, since the issue of genetic and, most importantly, morphological disunity between population groups in Asia, in the broad sense, and Melanesia remains unresolved. This issue must be left until more copious material collected in different areas of Asia appears, which would be studied by different methods, including the study of oil bodies in leaf cells. As it turned out over the course of the study, the genus *Gottschelia* could not be reliably assigned to any of the described families, and we proposed a new family to place this genus in the Cephaloziellaceae + Scapaniaceae macro-clade.

The geographical approach, widely used to describe the new-to-science species in the second half of the 19th and early 20th centuries, was undeservedly criticized in the middle of the 20th century. Since then, there has been a belief that liverworts have very wide species ranges. However, with the development of molecular genetic methods, it became necessary to admit that the use of a geographical approach in the taxonomy of liverworts is at times quite justified. The present account supports the effectiveness of the geographical approach, in which African populations represent one species and Asian populations represent another. There are many examples of species with admitted paleotropical ranges spanning from Africa to Melanesia. At the same time, it is not always reliably known whether this is actually one species or several. Further work on the interaction of genetic variability with geography will improve the understanding (and significance in general phytogeography) of a group of taxa with presumable paleotropical ranges.

## Figures and Tables

**Table 1:** The value of *p*-distances for specimens of the genus *Gottschelia* gathered in different geographical regions, %. “n/c”—Not calculated due to the presence of a single specimen, and “-”—Not calculated due to the absence of nucleotide sequence data.

Geographical Origin of the Specimens	*p*-Distances within Region, *rbc*L/*trn*L-F/*trn*G-intron, ITS1, ITS2, %	*p*-Distances between Regions, *rbc*L/*trn*L-F/*trn*G-intron, ITS1, ITS2, %			
		Vietnam	Malaysia–Sri Lanka	Indonesia	Madagascar–Reunion
Vietnam	0.0/0.0/0.0/0.0/0.0				
Malaysia–Sri Lanka	0.0/n/c/-/-/-	0.5/0.7/-			
Indonesia	0.0/-/-/-/-	0.9/-/-	0.3/-/-		
Madagascar–Reunion	0.0/-/n/c/-/-	2.1/-/6.0	2.0/-/-	1.9/-/-	
Tanzania	-/-/-/-/n/c	-/-/-/-/11.0	-/-/-/-/-	-/-/-/-/-	-/-/-/-/-

**Table 2:** The list of *Gottschelia* specimens sequenced in the current study with vouchers and GenBank accession numbers.

Specimen Voucher	GenBank Accession Number				
	ITS1 nrDNA	ITS2 nrDNA	*rbc*L cpDNA	*trn*L-F cpDNA	*trn*G-intron cpDNA
*Gottschelia microphylla* Vietnam: Nghe An Prov., V. Bakalin & M.H. Nguyen, V-88-6-23, 206354 (VBGI)	PP866642	PP864427	PP537608	PP537614	PP537611
*Gottschelia microphylla* Vietnam: Nghe An Prov., V. Bakalin & M.H. Nguyen, V-90-1-23, 206365 (VBGI)	PP866643	PP864428	PP537609	PP537615	PP537612
*Gottschelia microphylla* Vietnam: Nghe An Prov., V. Bakalin & M.H. Nguyen, V-100-3-23, 206518 (VBGI)	PP866644	PP864429	PP537610	PP537616	PP537613
*Gottschelia schizopleura* Tanzania, T. Pocs, J.S. Miala & P.P. Maragesi 90066/D No. 68 in BRYOPHYTA AFRICANA SELECTA Series III, Ochyra, Pocs (1992), 207901 (VBGI), 126285 (KPABG)	no data	PP864430	no data	no data	no data

**Table 3:** The list of specimens included in the molecular–phylogenetic analyses with voucher details and GenBank accession numbers.

Taxon	Specimen Voucher	GenBank Accession Number	
		*rbc*L cpDNA	*trn*L-F/*trn*G- intron cpDNA
*Adelanthus bisetulus*	New Zealand, Glenny 8858	DQ026580	DQ026614
*Alobiellopsis dominicensis*	Guadeloupe: Basse Terre, Schaefer-Verwimp & Verwimp 22133 (M)	KX098868	KX098946
*Anastrophyllopsis subcomplicata*	Australia, D. Meagher & N. Klazenga 07-142 (F)	KF943550	KF942968
*Anastrophyllum michauxii*	USA: North Carolina, M. Sargent s.n. (ABSH)	AY507390	AY507519
*Andrewsianthus australis*	Thailand, Schaefer-Verwimp & Verwimp 23734 (GOET)	KC184706	no data
*Andrewsianthus perigonialis*	New Zealand, J. Engel & M. von Konrat 27283 (GOET)	KC184707	no data
*Calypogeia arguta*	Japan: Kagoshima, Yakushima Isl., N. Hayashida 743 (CBM)	LC732076	LC732078
*Cephalozia hamatiloba*	Malaysia: Pahang, Schaefer-Verwimp & Verwimp 18559 (M)	KX098890	KX098968
*Cephaloziella divaricata*	Czech Republic, P. Sova (DUKE)	KF852399	KJ802094
*Cephaloziella hirta*	Australia, Streimann 59793 (NY)	DQ439682	KF943000
*Chaetophyllopsis whiteleggei*	Australia, J. Curnow 4804 (F)	KF852333	AY463553
*Cryptocolea imbricata*	Norway: Svalbard, A. Frisvoll 122667 (KPABG)	OR604355	OR604345
*Eremonotus myriocarpus*	United Kingdom: Scotland, B. Buryova 165/93 (DUKE)	KF852354	KJ802082
*Frullania longistipula*	Sao Tome and Principe: Sao Tome, Garcia 257,411 (LISU)	KU310979	KU310985
*Gottschelia schizopleura*	Indonesia, R. Gradstein 12065 (GOET)	FJ984941	no data
*Gottschelia schizopleura*	Indonesia, R. Gradstein 12064 (GOET)	FJ984942	no data
*Gottschelia schizopleura*	Malaysia: Pahang, D. Long 36922 (E)	KF852362	KJ802085
*Gottschelia schizopleura*	Malaysia, Schaefer-Verwimp 18695 (GOET)	FJ984944	no data
*Gottschelia schizopleura*	Sri Lanka, Schaefer-Verwimp 5488/II (GOET)	FJ984943	no data
*Gottschelia schizopleura*	Madagascar, Ah-Peng R96 (GOET)	FJ984940	no data
*Gottschelia schizopleura*	Reunion, Schaefer-Verwimp 19823 (GOET)	FJ984938	no data
*Gottschelia schizopleura*	Reunion, Gradstein 12032 (GOET)	FJ984939	no data
*Gottschelia schizopleura*	Reunion, T. Hedderson 15883 (BOL)	no data	no data/AM397736
*Gymnocolea borealis*	Russia: West Siberian Arctic, Gydansky Peninsula, E. Troeva G1-138 (LE)	MZ032229	MZ353627
*Gymnocolea fascinifera*	USA: Alaska, A. Potemkin, 92-9701 (LE)	MZ298896	MZ298895
*Gymnomitrion concinnatum*	Norway: Svalbard, N. Konstantinova & A. Savchenko K158/8-07 (F)	KF943557	KF942980
*Hattoria yakushimensis*	Japan: Kagoshima, Yakushima Island, T. Katagiri 4281 (NICH)	LC376047	LC376049
*Herzogianthus vaginatus*	New Zealand, Y. Qiu & J. Braggins NZ-03173 (AK)	DQ787462	no data
*Herzogobryum vermiculare*	French Southern and Antarctic Lands: Iles Kerguelen, R. Ochyra 1011/06 (DUKE)	KF943587	KF943047
*Jubula bogotensis*	Mexico, R. Gradstein s.n.	AY548100	DQ987388
*Jungermannia exsertifolia subsp. cordifolia*	USA: Alaska, B. Shaw F951/5 (DUKE)	KF943592	KF943051
*Konstantinovia beringii*	Russia: Kamchatka Terr., Commander Islands, Bering Isl., K. Klimova, Com-68-10-22 (VBGI)	OR785039	OR785035
*Konstantinovia pulchra*	China: Yunnan Prov., V. Bakalin & W.Z. Ma, C-77-2-18 (VBGI)	OR785040	MT476332
*Lejeunea cocoes*	Thailand, G. Lee 2496 (UKMB)	ON646222	ON646224
*Lepidolaena clavigera*	New Zealand, Frahm 22-3 (GOET)	EF545278	EF545373
*Liochlaena lanceolata*	Russia, 108000 (F)	KF943558	KF942981
*Lophozia ventricosa*	Finland, 2003 He-Nygren & Piippo 1473	AY462312	AY463572
*Marsupella sphacelata*	Russia, N. Konstantinova 29 June 2000 (F)	KF943561	KF942984
*Mylia anomala*	Canada: Alberta, D. Vitt s.n. (ABSH)	KF852367	KJ802088
*Neotrichocolea bissetii*	Japan, 1980 Inoue (5/03 200)	AY462317	AY463576
*Nothogymnomitrion erosum*	Australia, Streimann 53475 (JE), 1	GQ900318	GQ900216
*Nothogymnomitrion erosum*	New Zealand, J. Engel & M. von Konrat s.n. (F), 2	KF852369	KJ802089
*Nowellia curvifolia*	Germany: Bayern, Schaefer-Verwimp & Verwimp 28863 (M)	KX098919	KX099000
*Obtusifolium obtusum*	Switzerland: Canton of Valais, V. Bakalin, Sw-48-30-13 (VBGI)	OR785041	MT476337
*Odontoschisma denudatum*	Portugal: Azores, Schaefer-Verwimp & Verwimp 29511 (M)	KX098924	KX099005
*Oleolophozia perssonii*	Russia: Magadan Prov., V. Bakalin, Mag-31-13-11 (VBGI)	OR785042	MT476335
*Porella arboris-vitae*	Poland, J. Szweykowski 10429 (POZW)	KR014902	KR014871
*Ptilidium pulcherrimum*	Finland, Ahonen Engblom 7 (H)	AY302460	AY251186
*Scapania nemorea*	USA, Davis 124 (DUKE)	AY608039	AY608143
*Scapania undulata*	Finland, 2000 He-Nygren & Piippo 1468	AY149840	AY149859
*Schiffneria hyalina*	Japan, 1992 Mizutani 15961	AY462327	AY463585
*Solenostoma clavellatum*	Buthan, D. Long 28,636 (E)	KF943546	KF942951
*Southbya tophacea*	Russia: Krasnodar Terr., N. Konstantinova & A. Savchenko K312-4-11, 122707 (KPABG)	OR604354	OR604344
*Trilophozia quinquedentata*	Finland, 2003 He-Nygren & Piippo 1474	AY462334	AY463592
*Verdoornia succulenta*	New Zealand, Stotler & Crandall-Stotler 4602 (ABSH)	AY507430	AY507561
*Vietnamiella epiphytica*	Vietnam: Lao Cai Prov., V. Bakalin & K. Klimova V-9-7-17 (VBGI, KPABG)	MK290986	MK290984
*Wettsteinia inversa*	Indonesia, Gradstein 11014 (GOET)	FJ984935	GQ900275

## Data Availability

All data are contained within the article and Supplementary Materials.

## Supplementary Materials

The following supporting information can be downloaded at: https://www.mdpi.com/article/10.3390/plants13162198/s1, Table S1: Bioclimatic data obtained for *Gottschelia* specimens collected in Vietnam.
